# Mollicutes/HIV Coinfection and the Development of AIDS: Still Far from a Definitive Response

**DOI:** 10.1155/2016/8192323

**Published:** 2016-06-16

**Authors:** Caio Mauricio Mendes de Cordova, Caroline Galgowski, Leonardo Lange

**Affiliations:** ^1^Department of Pharmaceutical Sciences, University of Blumenau, 89010-350 Blumenau, SC, Brazil; ^2^Biomedical Sciences School, University of Blumenau, 89010-350 Blumenau, SC, Brazil

## Abstract

*Background*. Mycoplasmas are known to cause various infections in humans, mainly in the respiratory and urogenital tracts. The different species are usually host-specific and cause diseases in well-defined sites. New species have been isolated, including those from HIV-infected persons.* Summary*. Its* in vitro* properties, combined with clinical findings, have led to the hypothesis that these microorganisms may act as cofactors of HIV in AIDS development. Even today this point of view is quite polemic among infectious disease specialists and many aspects remain to be clarified, in contrast to what happens, for instance, with HIV/*Mycobacterium tuberculosis* coinfection. Dozens of papers have been published covering aspects of Mollicutes/HIV coinfection, but they add little to no information about the putative contribution of Mollicutes to the evolution of AIDS. Very few researchers have devoted their efforts to trying to answer this question, which remains open. In this review, we discuss the evidences that may support this statement in the light of current knowledge in the field of mycoplasmology.

## 1. Background

The incidence of sexually transmitted diseases (STDs) declined in the 1980s and early 1990s, reflecting changes in sexual behavior in response to the epidemic of HIV infection. But these changes were not maintained, and from the mid-1990s on there was a recurrence of sexually transmitted diseases. Pregnancy, birth at term, and fertility are affected by the presence of STDs, and it is crucial to investigate microorganisms such as* Chlamydia trachomatis*,* Neisseria gonorrhoeae*,* Candida* sp.,* Trichomonas vaginalis*, and mycoplasmas, or Mollicutes, in this situation [1]. Mycoplasmas and ureaplasmas are part of the class Mollicutes. Their most distinctive features are the absence of a cell wall and a reduced genome. They are a large group of microorganisms responsible for a number of diseases in animals, plants, and humans, especially sexually transmitted diseases [2].

For many years, Mollicutes species recognized as human pathogens of the genitourinary tract (TGU) were restricted to* Mycoplasma hominis* and* Ureaplasma urealyticum*, together with* Mycoplasma pneumoniae* in the respiratory tract. In the 1980s a new species of* Mycoplasma* was isolated from the urogenital tract of men with nongonococcal urethritis,* Mycoplasma genitalium*.* M. genitalium* is strongly associated with acute gonococcal urethritis, a condition in which it has an etiologic role now well recognized [3]. At the end of the 1980s,* M. fermentans*, a species isolated from the urogenital tract in the early 50s but considered infrequent in this site, was isolated from Kaposi's sarcoma lesions of a patient with AIDS and at that time classified as the* incognitus* strain of* M. fermentans*. Other cases of coinfection have been reported from peripheral blood mononuclear cells (PBMCs) and urine samples and demonstrated* M. fermentans* as the first invasive* Mycoplasma* in man [4].* M. penetrans* was also isolated from the urine of patients with AIDS. Its higher prevalence in these patients compared to HIV-negative individuals [5], together with its* in vitro* properties of being a strong activator of the immune system and stimulating the expression of HIV LTR-dependent genes, led* M. penetrans* to be suggested as a possible cofactor for HIV in the development of AIDS [6].

However, the relationship between Mollicutes infection and a possible worsening in the progression of AIDS has not yet been properly established. Dozens of publications cover aspects of Mollicutes/HIV coinfection, but they add little to no information about the putative contribution of Mollicutes to the evolution of AIDS, evaluating mostly the relative risk and factors associated with the coinfection. Very few researchers have devoted their efforts to trying to answer this question, which remains open. Contributing to these difficulties is the small number of researchers devoted to human clinical mycoplasmology around the world and the low appeal of urogenital tract Mollicutes infections to funding agencies. In addition, studies have shown that the detection of emerging Mollicutes species (*M. genitalium*,* M. fermentans*, and* M. penetrans*) only shows positive results with the use of molecular methods such as Polymerase Chain Reaction (PCR) [7].

The investigation of emerging* Mycoplasma* species infection and its possible relationship with the progression of AIDS in HIV-infected individuals can have important impacts on the quality of life of these individuals. Understanding this host/parasite relationship can contribute to better management of these infections and potentially reduce their morbidity.

## 2. Mollicutes as STD Agents

Mycoplasmas are the smallest microorganisms capable of self-replication. They are part of class Mollicutes, a large group of peculiar microorganisms whose main features are the absence of a cell wall and a reduced genome. Responsible for a number of diseases in animals, plants, and humans, they cause mainly sexually transmitted diseases (STDs) and respiratory infections [2]. However, atypical infections have been reported with increasing frequency, in either immunocompetent individuals or immunocompromised patients.

There are three species of Mollicutes worth mentioning in the context of urogenital tract infections:* Mycoplasma hominis*,* Ureaplasma urealyticum*, and* M. genitalium*, mainly acquired through sexual contact. All three have been found in male as well as female upper and lower urogenital tract infections.

*U. urealyticum* is responsible for cases of nongonococcal urethritis and bacterial vaginosis. Infection by the microorganism is associated with postpartum fever, chorioamnionitis, low birth weight and premature delivery, and complications in newborns.* U. urealyticum* was classically divided into 14 serotypes and grouped into two serogroups. More recently a new species has been defined,* U. parvum*, which includes the serotypes belonging to serogroup 1 [8].* M. hominis* is a cause of nongonococcal urethritis and bacterial vaginosis associated with postabortion and postpartum fever, epididymitis, and infertility. It is also an uncommon cause of bacteremia, endocarditis, arthritis, and other less common infections [9]. Several studies have shown that the rates of colonization by* M. hominis* and* U. urealyticum* in men range from zero to 13% and 3% to 56%, respectively. Similar data on women show that vaginal colonization rates by* M. hominis* and* U. urealyticum* range from zero to 31% and 8.5% to 77.5%, respectively, according to age, race, sexual experience, and socioeconomic status. A high incidence of infection by these microorganisms in Brazil, for instance, has also been demonstrated [10, 11].

For many years, the Mollicutes species recognized as human genitourinary tract (GUT) pathogens were restricted to* M. hominis* and* U. urealyticum* and* M. pneumoniae* in the respiratory tract. Beginning in the 1980s, this situation began to change with the isolation of a new* Mycoplasma* species,* M. genitalium*, from the urogenital tract of men with nongonococcal urethritis.* M. genitalium* was detected by PCR more often than in urethral specimens from men with nongonococcal urethritis compared to people without such symptoms, and anti-*M. genitalium* antibodies were more frequently detected in the sera of men with a positive PCR for this* Mycoplasma* than in sera from men with a negative PCR result. These data suggested that* M. genitalium* was strongly associated with acute nongonococcal urethritis, a hypothesis confirmed by several other studies and also its detection by PCR in samples from men with persistent or recurrent urethral disease followed by an acute attack of the disease, which today is well recognized [3].

### 2.1. *Mycoplasma fermentans*

In the late 1980s,* M. fermentans*, a species isolated from the urogenital tract in the early 50s but considered infrequent in this site, was isolated from Kaposi's sarcoma lesions of a patient with AIDS and finally classified as the* incognitus* strain of* M. fermentans* [4, 12]. Other isolates from HIV-infected patients have followed, from mononuclear cells of peripheral blood and urine samples, and demonstrated* M. fermentans* as the first invasive* Mycoplasma* in man [13]. These data, together with the observation that* M. fermentans* and other mycoplasmas can induce the replication of HIV* in vitro*, led Luc Montagnier, discoverer of HIV, and Alain Blanchard, renowned mycoplasmologist, for the first time to the hypothesis that these microorganisms could have a role in the development of AIDS [14].* M. fermentans* was also detected in patients with severe respiratory disease, sometimes fatal, especially in immunocompromised individuals, in which it may be responsible for more invasive conditions such as kidney and respiratory infections [15].* M. fermentans* was detected by PCR in the urine of HIV-infected homosexual men and in mononuclear cells from peripheral blood and throat samples with rates of 5, 10, and 20%, respectively. However, the same rates were found in HIV-negative individuals, most of them being homosexuals [16]. Thus, the importance of* M. fermentans* as a possible cofactor of HIV remained unsupported, coupled with no differences in detection in individuals with AIDS in different stages of the disease [17].

*M. fermentans* has also been isolated from throat samples of about 16% of children with pneumonia, being the only microorganism in two-thirds of the individuals [18]. It has been detected in some adults with an influenza-like illness, which in some cases has progressed rapidly, evolving to a respiratory disease, often fatal [19]. Observations on empirical treatments, including those by our group, tend to indicate that respiratory infections in children by* Mycoplasma*, probably by* M. fermentans*, are more common than it may seem and largely undiagnosed due to the lack of clinical suspicion and laboratory resources capable of identifying this microorganism. On the other hand,* M. fermentans* was detected by PCR in throat samples and urine in about 20% of healthy medical students, raising the question of what its preferred site of colonization and its importance in the etiology of diseases are [20]. In addition, to date, there is no evidence that* M. fermentans* is a cause of nongonococcal urethritis in men or disease in the urogenital tract of women [21]. Our experience has also shown a lack of isolation of* M. fermentans* in patients with urethritis, and we have detected its presence by PCR in only 0.9% of patients with symptoms, against 5.7% in HIV-infected individuals without symptoms of urethritis [5]. There is therefore a suspicion that under immunosuppressive conditions* M. fermentans* may act as an opportunistic pathogen, since it was also detected by PCR in bronchoalveolar lavage samples from 25% of people with AIDS presenting pneumonia, which was not the case in HIV-negative individuals [22]. The lack of consensus on the role of* M. fermentans* in the etiology of diseases demonstrates the complexity of the behavior of this agent and that many studies are still required to finally elucidate its real pathogenicity, which to this day remains a paradox.

### 2.2. *Mycoplasma penetrans*

*M. penetrans* is a species identified relatively recently as infecting man. It is also primarily isolated from the urine of patients with AIDS. Its seroprevalence in HIV-infected individuals was higher compared to HIV-negative individuals [7]. Along with its* in vitro* properties of being a potent activator of the immune system and stimulating HIV gene expression,* M. penetrans* holds a position as the best possible candidate for a cofactor of HIV in the development of AIDS [6].* M. penetrans* has the ability to induce an* in vitro* proliferative response of CD4 and CD8 T cells with expression of some activation markers such as CD69, HLA-DR, and CD 25 [23]. This finding is significant, considering the fact that HIV replicates completely only in activated cells. Still, in the context of modulating the immune response, it was observed that* M. penetrans* and* M. fermentans* membrane components can activate,* in vitro*, HIV LTR-dependent region gene expression [24]. It was suspected that this activation was due to the accumulation of oxygen free radicals after infection by* Mycoplasma*, since it has been shown that this accumulation results in an increase in NF-*κ*B, a known activator of the HIV LTR region [25]. It has been shown that activation of the HIV LTR is mediated by the interaction of* M. penetrans* membrane lipoproteins with Toll-like receptor-dependent NF-*κ*B [26]. Furthermore,* M. penetrans* has the ability to induce the production of TNF-alpha and HIV replication in cell lines [27]. Some authors have suggested an association between infection by* M. penetrans* and the progression of AIDS based on clinical study data. An early serological study in the USA showed the presence of anti-*M. penetrans* antibodies in 20% of asymptomatic HIV-positive individuals and in 40% of AIDS patients, compared to 0.3% in HIV-negative individuals [28]. In France, the prevalence of anti-*M. penetrans* was lower compared to previous reports, 18% in HIV-positive individuals and 1.3% in HIV-negative individuals [29]. In a study conducted in Africa, a different profile was found, with a 13.5% seroprevalence for* M. penetrans* in HIV-positive individuals and 15.5% in HIV-negative blood donors [30]. In that study, 25% of HIV-positive individuals with less than 5% CD4 cells had anti-*M. penetrans* antibodies, compared to 8.5% of subjects with more than 5% CD4 cells. In a longitudinal study in which adult HIV-positive homosexual men were followed for about 38 months, the association of the prevalence of anti-*M. penetrans* antibodies with the stage of AIDS was recorded; seroprevalence was twofold higher in patients in stage C of disease, according to the CDC classification at the time, than in stage A or B and twofold higher in individuals with a CD4: CD8 ratio less than 0.30 [31]. Of the studied subjects, 62% were seronegative for* M. penetrans*, 24% showed evidence of permanently low antibody levels or negative results in some cases, probably associated with latent or chronic infection, and 13.8% had evidence of moderate to high antibody levels for long periods, indicating an active and persistent* M. penetrans *infection. Four of these patients (50%) experienced serological reactivation, probably developing an acute infection during the study. The CD4+ cell count decreased significantly faster in the latter group than in those without anti-*M. penetrans* antibodies; the same behavior was observed in the group of individuals with low levels of antibodies. In individuals who had serological reactivation, the viral load was higher in serum from individuals with higher anti-*M. penetrans* antibody titers. These data suggested an association of active infection by* M. penetrans* with the progression of AIDS.

A higher prevalence of classes IgA and IgM anti-*M. penetrans* has also been demonstrated in HIV-infected individuals compared to the control group, detected by ELISA and confirmed by immunoblot [7]. Considering only IgA, the frequency of anti-*M. penetrans* antibodies was four times higher in individuals with a CD4: CD8 ratio lower than 0.3 (20% versus 5%). The presence of anti-*M. penetrans* IgM and IgA may be an indicator of a recent or active infection by the microorganism, especially in HIV-positive individuals with a lower CD4: CD8 ratio. Thus, there is evidence that an active* M. penetrans* infection may participate in the CD4 cell depletion process in HIV-infected individuals.

## 3. Mollicutes and AIDS Relationship

The growing knowledge about the biology of the classical* Mycoplasma* species and of new emerging ones, probably as opportunistic agents, has opened new horizons in human mycoplasmology. A case of infection by a species of* Mycoplasma* similar to* M. haemofelis*, which infects cats, has been observed in a subject with HIV [32].* M. pneumoniae*, a known pathogen of the respiratory tract, may also present a more complicated infection in patients with AIDS [33]. However, the parasite-host relationship in individuals with HIV coinfected by mycoplasmas still presents significant gaps. A better understanding of this relationship may have major implications for the quality of life of infected individuals, preventing, for example, the development of systemic infections, possibly lethal, as has been observed at least with* M. fermentans*. Even better known* Mycoplasma* species such as* M. hominis* and* Ureaplasma urealyticum*/*parvum* can be isolated from individuals infected with HIV at higher rates than from HIV-negative individuals [10]. In fact, a number of microorganisms have been proposed as potential HIV cofactors in the development of AIDS, such as* Leishmania*, mycobacteria, and viruses [6]. However, the chronic nature of their infection, their greater prevalence in patients with AIDS, and* in vitro* studies of its properties have positioned the mycoplasmas, in particular* M. penetrans*, as the microorganisms that seem to have the greatest potential to act as possible cofactors in the development of HIV/AIDS [34]. At that time, this hypothesis was discredited by most virologists, who believed that HIV infection per se would be sufficient to cause immunosuppression. On the other hand, it has received more attention by a number of immunologists, considering the complexity of the immune response against viral infection when specifically targeting T-lymphocytes. It is worth noting that the virus entry into the cell occurs more efficiently after activation of the immune system, when there is greater expression of CD4 molecules, recognized as the main receptor used by HIV for cell adhesion and invasion [35]. However, this issue is far from achieving a consensus among mycoplasmologists. For example, Ainsworth et al. found no difference in the presence of* M. fermentans* in patients seropositive for HIV regardless of whether they were nonprogressors, slow progressors, or rapid progressors [36]. These authors also found no* M. penetrans* DNA in their samples.

This subject is still very controversial, among infectious diseases specialists and even among mycoplasmologists. For* Mycobacterium tuberculosis*, for instance, there is practically a consensus that, besides being an opportunistic pathogen, the bacterium acts as a cofactor for HIV/AIDS, worsening its progression [37, 38]. It is believed that* M. tuberculosis* affects the progression of AIDS, mainly by activation of the immune system and by increased expression of CCR5 and CXCR4 [39, 40]; however, apparently the presence of the bacterial infection does not increase the viral load [41], whereas,* in vitro*,* M. tuberculosis* seems to actually reduce viral replication [42]. Furthermore, HIV infects* M. tuberculosis*-specific CD4+ T cells with a Th17 polarization profile, what may explain an exacerbation of tuberculosis in HIV-infected subjects [43]. For additional information about the details of immune responses to TB and HIV coinfection please refer to the review by Shankar et al. [44].

In the same way, additional studies would be very welcome to shed more light regarding HIV/Mollicutes coinfection, generating new insights into understanding this complex host-parasite relationship. Little to no further significant advance has been made in the recent years to elucidate the putative mechanisms through which Mollicutes would interact with the immune system that could worsen the HIV infection, especially considering the abovementioned* Mycoplasma* species. Only for* M. pneumoniae* pneumonia, a primary human pathogen for which an animal model of disease is also well stablished, interactions of the microorganism with the immune system are better understood [45].

## 4. Recent Findings

A study was recently conducted trying to evaluate a possible correlation between Mollicutes infection and AIDS progression over a 3.5-year period. In this population, 30 individuals presented a positive result for urogenital Mollicutes (37.5%) and 50 were negative (62.5%) by culture. No difference was observed in Mollicutes positivity between men and women. Among the positive samples, 21 presented* Ureaplasma* sp. growth, 23 presented* M. hominis *growth, and 7 presented Mollicutes growth in SP4 medium.* M. penetrans* was identified in these samples by PCR. No difference (*P* > 0.05) was observed in CD4 or CD8 cells levels or the CD4/CD8 relationship between Mollicutes infected and noninfected patients. However, patients infected by Mollicutes presented a higher HIV viral load during the study period (*P* = 0.007) compared to noninfected patients. A difference in opportunistic/coinfection incidence between the Mollicutes infected and the noninfected group was also observed [46].* M. genitalium* was also observed to be strongly associated with HIV infection in several isolated studies and by a meta-analysis [47].* U. urealyticum*,* M. hominis*, and* M. fermentans* were also found to be associated with HIV patients by other groups [48]. However, association does not indicate a causal effect, and only a randomized controlled trial, for instance, detecting and treating a group of Mollicutes infected individuals with antibiotics while leaving another group to naturally progress during HIV disease, may clearly answer this question. One of the major questions to be answered is if any Mollicutes infection is treated, does it result in a decrease in viral load among HIV patients or stop/delay progression to AIDS? This would be an audacious approach in the current times we live in regarding ethical issues, but a brave research group with the support of a well-established HIV clinic willing to conduct such study would be very welcome and would leave its permanent mark in elucidating the role of Mollicutes/HIV coinfections in the development of AIDS.

Contrary to what occurs in coinfection with* M. tuberculosis*, Mollicutes increase virus replication,* in vitro* and probably also* in vivo* [14]. The higher HIV viral load in patients infected with Mollicutes is probably due to this property, as stated at least for* M. penetrans* and* M. fermentans*, to induce viral replication [6]. Regardless of the Mollicutes species presence, the coinfection seems to contribute to the replication of HIV. However, so far there is only controversial evidence for increased degradation of the immune system in patients infected with Mollicutes. In our study, Mollicutes infected patients showed no increased incidence of opportunistic disease compared with uninfected patients [46]. Therefore, these findings do not corroborate a possible hypothesis that mycoplasmas could simply be opportunistic agents during an HIV infection. In conclusion, over three and a half years of study, it was demonstrated that HIV patients coinfected with urogenital Mollicutes have a higher viral load than patients without Mollicutes. Additional studies evaluating a longer period of time could provide more precise information as to the possibility of a more rapid degradation of the immune system in these individuals and its relation to Mollicutes infection.

## References

[1] Jaiyeoba O., Lazenby G., Soper D. E. (2011). Recommendations and rationale for the treatment of pelvic inflammatory disease. *Expert Review of Anti-Infective Therapy*.

[2] Razin S., Yogev D., Naot Y. (1998). Molecular biology and pathogenicity of mycoplasmas. *Microbiology and Molecular Biology Reviews*.

[3] Bradshaw C. S., Tabrizi S. N., Read T. R. H. (2006). Etiologies of nongonococcal urethritis: bacteria, viruses, and the association with orogenital exposure. *Journal of Infectious Diseases*.

[4] Lo S.-C., Dawson M. S., Wong D. M. (1989). Identification of Mycoplasma incognitus infection in patients with AIDS: an immunohistochemical, in situ hybridization and ultrastructural study. *American Journal of Tropical Medicine and Hygiene*.

[5] Cordova C. M. M., Blanchard A., Cunha R. A. F. (2000). Higher prevalence of urogenital mycoplasmas in human immunodeficiency virus-positive patients as compared to patients with other sexually transmitted diseases. *Journal of Clinical Laboratory Analysis*.

[6] Blanchard A. (1997). Mycoplasmas and HIV infection, a possible interaction through immune activation. *Wiener Klinische Wochenschrift*.

[7] de Cordova C. M. M., Takei K., Rosenthal C., Miranda M. A. R. B., Vaz A. J., da Cunha R. A. F. (1999). Evaluation of IgG, IgM, and IgA antibodies to *Mycoplasma penetrans* detected by ELISA and immunoblot in HIV-1-infected and STD patients, in Sao Paulo, Brazil. *Microbes and Infection*.

[8] Glass J. I., Lefkowitz E. J., Glass J. S., Heiner C. R., Chen E. Y., Cassell G. H. (2000). The complete sequence of the mucosal pathogen Ureaplasma urealyticum. *Nature*.

[9] González-Jiménez M. A., Villanueva-Díaz C. A. (2006). Epididymal stereocilia in semen of infertile men: evidence of chronic epididymitis?. *Andrologia*.

[10] Cordova C. M. M., Cunha R. A. F. (2000). Relevant prevalence of *Mycoplasma hominis* and *Ureaplasma urealyticum* serogroups in HIV-1 infected men without urethritis symptoms. *Revista do Instituto de Medicina Tropical de São Paulo*.

[11] Miranda Filho J. C., Dalmarco E. M., de Cordova C. M. (2004). Mycoplasma and ureaplasma infection in women. *Dynamis*.

[12] Sasaki T., Sasaki Y., Kita M., Suzuki K., Watanabe H., Honda M. (1992). Evidence that Lo's mycoplasma (Mycoplasma fermentans incognitus) is not a unique strain among Mycoplasma fermentans strains. *Journal of Clinical Microbiology*.

[13] Barré-Sinoussi F., Chermann J. C., Rey F. (1983). Isolation of a T-lymphotropic retrovirus from a patient at risk for acquired immune deficiency syndrome (AIDS). *Science*.

[14] Montagnier L., Blanchard A. (1993). Mycoplasmas as cofactors in infection due to the human immunodeficiency virus. *Clinical Infectious Diseases*.

[15] Ainsworth J. G., Hourshid S., Clarke J. (1994). Detection of Mycoplasma fermentans in HIV-positive individuals undergoing bronchoscopy. *IOM Letters*.

[16] Katseni V. L., Ryait B. K., Ariyoshi K. (1993). Mycoplasma fermentans in individuals seropositive and seronegative for HIV-1. *The Lancet*.

[17] Taylor-Robinson D. (1996). Infections due to species of *Mycoplasma* and *Ureaplasma*: an update. *Clinical Infectious Diseases*.

[18] Cassel G. H., Yanez A., Duddy L. B. (1994). Detection of Mycoplasma fermentans in the respiratory tract of children with pneumonia. *IOM Letters*.

[19] Lo S., Kotani H., Hu W. S. (1993). Molecular and cell biology of opportunistic infections in AIDS. Mycoplasmas and AIDS. *Molecular and Cell Biology of Human Diseases Series*.

[20] Taylor-Robinson D., Furr P. M. (1997). Genital mycoplasma infections. *Wiener Klinische Wochenschrift*.

[21] Deguchi T., Gilroy C. B., Taylor-Robinson D. (1996). Failure to detect *Mycoplasma fermentans*, *Mycoplasma penetrans*, or *Mycoplasma pirum* in the urethra of patients with acute nongonococcal urethritis. *European Journal of Clinical Microbiology and Infectious Diseases*.

[22] Ainsworth J. G., Katseni V., Hourshid S. (1994). Mycoplasma fermentans and HIV-associated nephropathy. *Journal of Infection*.

[23] Sasaki Y., Blanchard A., Watson H. L. (1995). In vitro influence of Mycoplasma penetrans on activation of peripheral T lymphocytes from healthy donors or human immunodeficiency virus-infected individuals. *Infection and Immunity*.

[24] Nir-paz R., Israel S., Honigman A., Kahane I. (1995). Mycoplasmas regulate HIV-LTR-dependent gene expression. *FEMS Microbiology Letters*.

[25] Gaynor R. (1992). Cellular transcription factors involved in the regulation of HIV-1 gene expression. *AIDS*.

[26] Shimizu T., Kida Y., Kuwano K. (2004). Lipid-associated membrane proteins of *Mycoplasma fermentans* and *M. penetrans* activate human immunodeficiency virus long-terminal repeats through Toll-like receptors. *Immunology*.

[27] Iyama K., Ono S., Kuwano K., Ohishi M., Shigematsu H., Arai S. (1996). Induction of tumor necrosis factor alpha (TNF*α*) and enhancement of HIV-1 replication in the J22HL60 cell line by *Mycoplasma penetrans*. *Microbiology and Immunology*.

[28] Wang R. Y.-H., Hayes M. M., Wear D. J. (1992). High frequency of antibodies to Mycoplasma penetrans in HIV-infected patients. *The Lancet*.

[29] Grau O., Slizewicz B., Tuppin P. (1995). Association of Mycoplasma penetrans with human immunodeficiency virus infection. *Journal of Infectious Diseases*.

[30] Tuppin P., Delamare O., Launay V. (1997). High prevalence of antibodies to Mycoplasma penetrans in human immunodeficiency virus-seronegative and -seropositive populations in Brazzaville, Congo. *Clinical and Diagnostic Laboratory Immunology*.

[31] Grau O., Tuppin P., Slizewicz B. (1998). A longitudinal study of seroreactivity against Mycoplasma penetrans in HIV-infected homosexual men: association with disease progression. *AIDS Research and Human Retroviruses*.

[32] Dos Santos A. P., Dos Santos R. P., Biondo A. W. (2008). Hemoplasma infection in HIV-positive patient, Brazil. *Emerging Infectious Diseases*.

[33] Watson M. E., Storch G. A. (2008). Recurrent mycoplasma pneumoniae infection in a human immunodeficiency virus-positive child. *Pediatric Infectious Disease Journal*.

[34] Brenner C., Neyrolles O., Blanchard A. (1996). Mycoplasmas and HIV infection: from epidemiology to their interaction with immune cells. *Frontiers in Bioscience*.

[35] Cullen B. R., Greene W. C. (1989). Regulatory pathways governing HIV-1 replication. *Cell*.

[36] Ainsworth J. G., Hourshid S., Easterbrook P. J., Gilroy C. B., Weber J. N., Taylor-Robinson D. (2000). Mycoplasma species in rapid and slow HIV progressors. *International Journal of STD and AIDS*.

[37] Badri M., Ehrlich R., Wood R., Pulerwitz T., Maartens G. (2001). Association between tuberculosis and HIV disease progression in a high tuberculosis prevalence area. *International Journal of Tuberculosis and Lung Disease*.

[38] López-Gatell H., Cole S. R., Hessol N. A. (2007). Effect of tuberculosis on the survival of women infected with human immunodeficiency virus. *American Journal of Epidemiology*.

[39] Vanham G., Edmonds K., Qing L. (1996). Generalized immune activation in pulmonary tuberculosis: co-activation with HIV infection. *Clinical and Experimental Immunology*.

[40] Wolday D., Tegbaru B., Kassu A. (2005). Expression of chemokine receptors CCR5 and CXCR4 on CD4+ T cells and plasma chemokine levels during treatment of active tuberculosis in HIV-1-coinfected patients. *Journal of Acquired Immune Deficiency Syndromes*.

[41] Srikantiah P., Wong J. K., Liegler T. (2008). Unexpected low-level viremia among HIV-infected Ugandan adults with untreated active tuberculosis. *Journal of Acquired Immune Deficiency Syndromes*.

[42] Goletti D., Carrara S., Vincenti D. (2004). Inhibition of HIV-1 replication in monocyte-derived macrophages by *Mycobacterium tuberculosis*. *Journal of Infectious Diseases*.

[43] Saharia K. K., Koup R. A. (2013). XT cell susceptibility to HIV influences outcome of opportunistic infections. *Cell*.

[44] Shankar E. M., Vignesh R., Ellegård R. (2014). HIV-Mycobacterium tuberculosis co-infection: a ‘danger-couple model’ of disease pathogenesis. *Pathogens and Disease*.

[45] Saraya T., Kurai D., Nakagaki K. (2014). Novel aspects on the pathogenesis of *Mycoplasma pneumoniae* pneumonia and therapeutic implications. *Frontiers in Microbiology*.

[46] Sampaio J., Ceola C. F., Machado L. D. P. N. (2013). Assessment of immunosuppression progress in patients with HIV/AIDS in the presence of urogenital tract mycoplasmas. *Tendencias em HIV/AIDS*.

[47] Mavedzenge S. N., Weiss H. A. (2009). Association of *Mycoplasma genitalium* and HIV infection: a systematic review and meta-analysis. *AIDS*.

[48] Wang B., Wu J.-R., Guo H.-J. (2012). The prevalence of six species of *Mycoplasmataceae* in an HIV/AIDS population in Jiangsu Province, China. *International Journal of STD and AIDS*.

